# Serum Adhesion Molecule Levels as Prognostic Markers in Patients with Early Systemic Sclerosis: A Multicentre, Prospective, Observational Study

**DOI:** 10.1371/journal.pone.0088150

**Published:** 2014-02-06

**Authors:** Minoru Hasegawa, Yoshihide Asano, Hirahito Endo, Manabu Fujimoto, Daisuke Goto, Hironobu Ihn, Katsumi Inoue, Osamu Ishikawa, Yasushi Kawaguchi, Masataka Kuwana, Fumihide Ogawa, Hiroki Takahashi, Sumiaki Tanaka, Shinichi Sato, Kazuhiko Takehara

**Affiliations:** 1 Department of Dermatology, School of Medicine, Faculty of Medical Sciences, University of Fukui, Fukui, Japan; 2 Department of Dermatology, School of Medicine, Institute of Medical, Pharmaceutical, and Health Sciences, Kanazawa University, Kanazawa, Japan; 3 Department of Dermatology, University of Tokyo Graduate School of Medicine, Bunkyo-ku, Tokyo, Japan; 4 Department of Internal Medicine (Omori), Toho University School of Medicine, Ota-ku, Tokyo, Japan; 5 Department of Dermatology, Faculty of Medicine, University of Tsukuba, Tsukuba, Ibaraki, Japan; 6 Department of Rheumatology, Faculty of Medicine, University of Tsukuba, Tsukuba, Ibaraki, Japan; 7 Department of Dermatology and Plastic Surgery, School of Life Sciences, Kumamoto University, Kumamoto, Kumamoto, Japan; 8 Division of Rehabilitation Science, Kanazawa University Graduate School of Medical Science, Kanazawa, Ishikawa, Japan; 9 Department of Dermatology, Gunma University Graduate School of Medicine, Maebashi, Gunma, Japan; 10 Institute of Rheumatology, Tokyo Women's Medical University, Shinjuku-ku, Tokyo, Japan; 11 Division of Rheumatology, Department of Internal Medicine, Keio University School of Medicine, Shinjuku-ku, Tokyo, Japan; 12 Department of Dermatology, Nagasaki University Graduate School of Biomedical Science, Nagasaki, Nagasaki, Japan; 13 Department of Gastroenterology, Rheumatology and Clinical Immunology, Sapporo Medical University School of Medicine, Sapporo, Hokkaido, Japan; 14 Department of Rheumatology and Infectious Diseases, Kitasato University School of Medicine, Sagamihara, Kanagawa, Japan; University of Texas Health Science Center at Houston, United States of America

## Abstract

**Objective:**

To assess the utility of circulating adhesion molecule levels as a prognostic indicator of disease progression in systemic sclerosis (SSc) patients with early onset disease.

**Methods:**

Ninety-two Japanese patients with early onset SSc presenting with diffuse skin sclerosis and/or interstitial lung disease were registered in a multicentre, observational study. Concentrations of intercellular adhesion molecule (ICAM) −1, E-selectin, L-selectin, and P-selectin in serum samples from all patients were measured by enzyme-linked immunosorbent asssay (ELISA). In 39 patients, adhesion molecule levels were measured each year for four years. The ability of baseline adhesion molecule levels to predict subsequent progression and severity in clinical and laboratory features were evaluated statistically.

**Results:**

At their first visit, serum levels of ICAM-1, E-selection, P-selectin were significantly elevated and serum L-selectin levels were significantly reduced in patients with SSc compared with healthy controls. Overall, serum ICAM-1 levels at each time point were significantly inversely associated with the %vital capacity (VC) of the same time and subsequent years by univariate analysis. The initial serum ICAM-1 levels were significantly inversely associated with the %VC at the fourth year by multiple regression analysis. The initial serum P-selectin levels were significantly associated with the health assessment questionnaire disability index (HAQ-DI) at the fourth year by multiple regression analysis. Initial adhesion molecule levels were not significantly associated with other clinical features including skin thickness score. Baseline adhesion molecule levels were not significantly associated with subsequent rate of change of clinical parameters.

**Conclusion:**

In patients with SSc, serum levels of ICAM-1 and P-selectin may serve as prognostic indicators of respiratory dysfunction and physical disability, respectively. Further longitudinal studies of larger populations are needed to confirm these findings.

## Introduction

Systemic sclerosis (SSc) is a connective tissue disease characterized by tissue fibrosis in the skin and internal organs, and vascular involvement [1], [2]. Interstitial lung disease (ILD) develops in more than half of SSc patients and is one of the major SSc-related causes of death. Joint contracture due to extensive skin sclerosis and/or severe internal organ involvement results in impaired physical function.

SSc patients exhibit increased numbers and activation of monocytes/macrophages and T cells in the circulation and tissues [3], [4]. Infiltration of these cells into the skin or internal organs may promote endothelial damage and fibrosis, most likely through the production of soluble mediators including cytokines and chemokines. Leukocyte recruitment into inflammatory sites is generally achieved using multiple cell adhesion molecules [5].

E-selectin, (CD62E), L-selectin (CD62L), and P-selectin (CD62P) primarily mediate leukocyte capture and rolling on the endothelium [6]. L-selectin is constitutively expressed on most leukocytes [6]. Whereas P-selectin is rapidly mobilized to the surface of activated endothelium or platelets, E-selectin expression is induced within several hours after activation with inflammatory cytokines [6]. These selectins share a highly conserved N-terminal lectin domain that can interact with sialylated and fucosylated oligosaccharides such as sialyl Lewis X [7]. Although various candidates have been identified as potential ligands for selectins, P-selectin glycoprotein ligand 1 (PSGL-1) is the best characterized ligand, which is recognized by all three selectins [8). PSGL-1 is a mucin-like, disulfide-linked homodimer expressed by all subsets of leukocytes and is a high-affinity ligand for E- and P-selectins [9). PSGL-1 has also been shown to bind to L-selectin, but its affinity is lower than E- and P-selectins [10].

Intercellular adhesion molecule (ICAM)-1 (CD54) is a member of the Ig superfamily that is constitutively expressed not only on endothelial cells, but also on fibroblasts and epithelial cells [11]. It can be upregulated transcriptionally by several proinflammatory cytokines, such as interleukin (IL) -1, interferon (IFN) -γ, and tumor necrosis factor (TNF) –α [11]. ICAM-1 binds to leukocyte function associated antigen-1 (LFA-1) and macrophage adhesion ligand-1 (Mac-1). LFA-1 and Mac-1 expressed on leukocytes bind to ICAM-1 to mediate firm adhesion and transmigration of leukocytes across vascular endothelia in processes such as extravasation and the inflammatory response [5].

In most patients, severe organ involvement occurs within the first three years of disease and skin sclerosis seldom progresses after five or six years [12], [13]. Therefore, predicting disease progression is particularly important for SSc patients at their first visit. However, except for SSc-related autoantibodies [14] there are no definitive serum biomarkers available to estimate disease progression. We hypothesized that some adhesion molecules may be related to underlying biologic process which is ongoing and which will change clinical features over time.

In the present study, we focused on major 4 adhesion molecules (ICAM-1, E-selectin, L-selectin, and P-selectin). We sought to determine if baseline serum adhesion molecule levels could predict the progress of symptoms in early SSc patients.

## Methods

### Patients

Patients were grouped according to the degree of skin involvement based upon the classification system proposed by LeRoy *et al.* [diffuse cutaneous SSc (dcSSc) versus limited cutaneous SSc (lcSSc)] [15]. In this study, 92 Japanese patients with early SSc (disease duration defined by the period from the first symptom including Raynaud’s phenomenon attributable to SSc to our first assessment ≤ three years) who had dcSSc and/or ILD were registered at nine major scleroderma centers in Japan (Gunma University Hospital, Kanazawa University Hospital, Keio University Hospital, Kumamoto University Hospital, Nagasaki University Hospital, Tokyo University Hospital, Tokyo Women's Medical University Hospital, Toho University Omori Medical Center, Tsukuba University Hospital). Patients with other inflammatory, infectious, or malignant diseases were not included in this study.

Among the patients, 49 patients had dcSSc with ILD, 30 patients had dcSSc without ILD, and 13 patients had lcSSc with ILD. Sixty-four patients were female and twenty-eight patients were male. The median age was 53 (range, 14–76). The median disease duration was 19 months (range, 1–60 months). All patients fulfilled the criteria for SSc proposed by the American College of Rheumatology [16]. With respect to the specificity of anti-nuclear antibodies (Abs) in the serum, 56 patients were positive for anti-topoisomerase I Ab and 11 patients were positive for anticentromere Ab. Age and gender-matched 24 healthy persons (17 females and 7 males, median age 49 (range, 20–65) ) were also included as normal controls in this study.

Among 92 patients, 39 patients could be followed every year for four years. Twenty-three patients had dcSSc with ILD, seven patients had dcSSc without ILD, and nine patients had lcSSc with ILD. Twenty-seven patients were female and twelve patients were male. The median age was 54 (range, 14–75). The median disease duration was 20 months (range, 1–60). With respect to the specificity of anti-nuclear Abs, 25 patients were positive for anti-topoisomerase I Ab and three patients was positive for anticentromere Ab. The ethical committee at each centre (Institutional Review Board, Gunma University Hospital; Kanazawa University Ethical Committee; Keio University Ethical Committee; Ethics Committee for Clinical Research and Advanced Medical Technology at the Faculty of Life Sciences, Kumamoto University; Ethics Committee of Nagasaki University Hospital; the Ethical Committee of the Faculty of Medicine, University of Tokyo; the Ethics Committee of Tokyo Women's Medical University, the Ethics Committee of Toho University Omori Medical Center; Ethics Committee University of Tsukuba Hospital) approved all protocols and informed written consent was obtained from all patients.

### Clinical Assessments

Patients had a physical examination and laboratory tests were performed at their first visit and at each subsequent year for four years. The degree of skin involvement was determined according to the modified Rodnan total skin thickness score (MRSS), as described elsewhere [17]. Organ system involvement was defined as described previously [18] with some modifications: ILD = bibasilar interstitial fibrosis or ground-glass shadow on computed tomogram (CT); pulmonary arterial hypertension (PAH) = clinical evidence of pulmonary hypertension and elevated right ventricular systolic pressure (>45 mmHg) documented by echocardiography in the absence of severe pulmonary interstitial fibrosis; esophagus = apparent dysphagia, reflux symptoms, or hypomotility shown by barium radiography; heart = pericarditis, congestive heart failure, or arrhythmias requiring treatment; kidney = malignant hypertension and rapidly progressive renal failure unexplained by certain diseases other than SSc; joint = inflammatory polyarthralgias or arthritis; and muscle = proximal muscle weakness and elevated serum creatine kinase. A health assessment questionnaire-disability index (HAQ-DI) modified for Japanese patients [19] including digital ulcer, pitting scar, maximal oral aperture (the maximum vertical length of opened mouth), and skin pigmentation/depigmentation was also evaluated. Erythrocyte sedimentation rate (ESR) and pulmonary function including vital capacity (VC) were also tested.

### ELISA

Fresh venous blood samples were taken from 92 patients and 24 healthy controls at their first visit (baseline). In 39 patients, blood samples were also taken at each subsequent year for four years. Samples were centrifuged shortly after clot formation. All serum samples were stored at −70°C prior to use in assays. Serum levels of ICAM-1, E-selectin, L-selectin, and P-selectin were measured by ELISA (R&D systems, Inc. Minneapolis, MN). Limit of detection was as follows; ICAM-1 31.2 pg/ml, E-selectin 93.8 pg/ml, L-selectin 78.1 pg/ml, and P-selectin 125 pg/ml.

### Statistical Analysis

JMP^®^ Statistical Discovery Software (SAS Institute, Cary, NC) was used for analysis. Since Shapiro-Wilk test did not indicate that serum adhesion molecule concentration showed normal distribution, the data were converted to logarithm so that the data exhibited normal distribution. Then, statistical analyses were performed using the t-test for the comparison of sample levels between two groups. The Pearson product-moment correlation coefficient was used to examine the relationship between two continuous variables. Potential prognostic factors for estimating subsequent MRSS, %VC, and HAQ-DI were statistically examined by multiple regression analysis. A p-value <0.05 was considered statistically significant. All values are expressed as the median (range) otherwise indicated.

## Results

### Serum Levels of Adhesion Molecules were Elevated in SSc Patients

**Figure 1:**
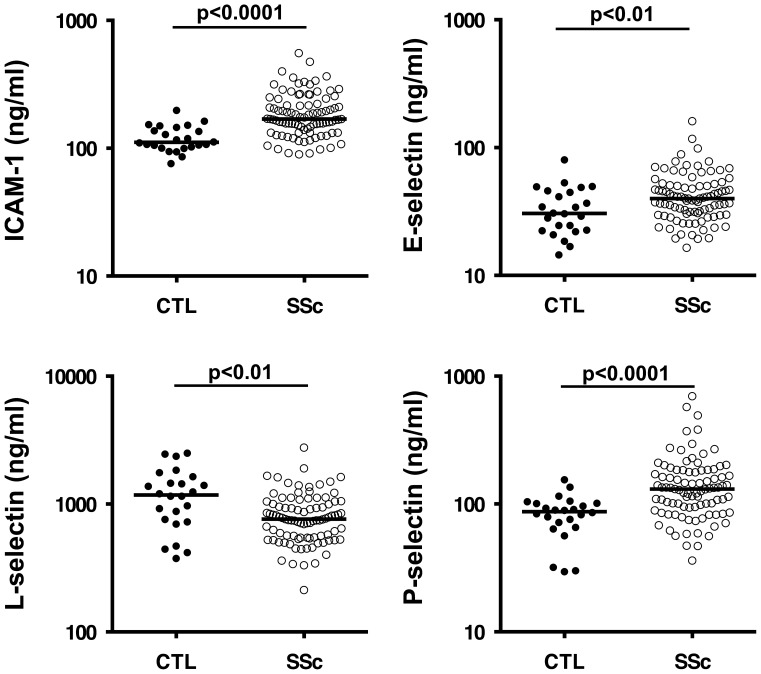
Serum adhesion molecule levels in healthy controls (CTL) and early systemic sclerosis (SSc) patients with diffuse skin sclerosis and/or interstitial lung diseases. The horizontal bar in each group indicates the median value.

Serum samples were taken from normal controls (n = 24) and all patients (n = 92) at their first visit. Serum levels of ICAM-1 were significantly increased in SSc patients compared with healthy controls (p<0.0001, Figure 1). Serum levels of E-selectin and P-selectin were also significantly elevated in the SSc patients (p<0.01 vs. p<0.0001, respectively, Figure 1). By contrast, serum L-selectin levels were significantly reduced in patients with SSc (p<0.01, Figure 1). Serum levels of ICAM-1 were significantly associated with levels of E-selectin in patients (r = 0.51, p<0.0001). However, other combinations of adhesion molecules were not significantly associated with each other.

At the initial visit, serum ICAM-1 levels were significantly elevated in patients with ILD compared with patients without it (median (range) ng/ml; 179.3 (91.6–556.7) vs. 165.9 (89.7–263.5), p<0.05). There were significant inverse associations between serum ICAM-1 levels and %VC in patients with SSc (r = −0.41 p<0.001). In addition, serum ICAM-1 levels were significantly elevated in patients with anti-topoisomerase I Ab than in patients without it (median (range) ng/ml; 183.5 (104.9–556.7) vs. 156.5 (89.7–331.1), p<0.01). There was also a significant association between serum P-selectin levels and HAQ-DI (r = 0.30, p<0.01). However, no significant correlations were found between the levels of any of the adhesion molecules measured and any other clinical or laboratory findings. Steroid treatment did not significantly affect the levels of these adhesion molecules (steroid (+) vs. steroid (−) (median (range) ng/ml); ICAM-1 160.8 (91.1–556.7) vs. 170.0 (89.7–474.8), p = 0.14; E-selectin 41.2 (19.3–161.0) vs. 36.1 (16.4–117), p = 0.22; L-selectin 800.5 (213.2–3989.7) vs. 745.7 (343.2–1423.9), p = 0.62; P-selectin 132.8 (36.2–699.5) vs. 122.6 (56.2-492.8), p = 0.53.

### Longitudinal change of Clinical Features

To assess progression of SSc over time, clinical features of thirty-nine patients who were able to be followed-up every year for four years were analyzed (Table 1). To assess the degree of skin involvement in patients, MRSS values were calculated, and %VC was used to assess lung involvement. HAQ-DI was also obtained in order to evaluate the functional abilities of the patients. For the patient population as a whole, the median MRSS value decreased from 16 to 10 during the first year. The median MRSS was 12 at the end of year two, 9 at the end year three, and 8 at the end year four. Median values for %VC did not significantly change during the four-year evaluation period. In this regard, the %VC was 96 at first visit, 91 at the end of the first year, 95 at the end of the second year, 91 at the end of the third year, and 90 at the end of the fourth year. The median HAQ-DI was 0.125 at the first visit and at the end of year one and three, whereas it was 0.25 at the end of year two and four. ILD and renal crisis were newly detected during the evaluation period in 2 and 4 patients, respectively. No patients had PAH during the period. Most patients were treated with oral prednisolone during the follow-up period. Additionally, a part of patients were treated with immunosuppresive agents including cyclophosphamide, cyclosporin A, azathioprine, and methotrexate.

**Table 1:** The course of clinical and laboratory features in patients with SSc.

	Baseline	1 year follow-up	2 year follow-up	3 year follow-up	4 year follow-up
MRSS	16 (2–39)	10 (0–38)	12 (0–35)	9 (1–25)	8 (0–29)
And meto	96 (53–143)	91 (62–143)	95 (61–143)	91 (56–137)	90 (58–136)
HAQ-DI	0.125 (0–1.5)	0.125 (0–1.75)	0.25 (0–2.5)	0.125 (0–1.875)	0.25 (0–1.75)
ILD	30 (77%)	30 (77%)	31 (79%)	32 (82%)	32 (82%)
Renal crisis	0 (0%)	2 (5.1%)	0 (0%)	1 (2.6%)	1 (2.6%)
Corticosteroid therapy	26 (67%)	32 (82%)	33 (85%)	34 (87%)	32 (82%)
Cyclophosphamide therapy	4 (10%)	8 (21%)	4 (10%)	6 (15%)	8 (21%)
Cyclosporin A therapy	0 (0%)	1 (3%)	2 (5%)	4 (10%)	4 (10%)
Azathioprine therapy	0 (0%)	0 (0%)	1 (3%)	1 (3%)	1 (3%)
Methotrexate therapy	0 (0%)	0 (0%)	0 (0%)	0 (0%)	1 (3%)

Values are represented as median (range) or as number of positive cases with percentage within parentheses.

### Longitudinal Change of Adhesion Molecule Levels

**Figure 2:**
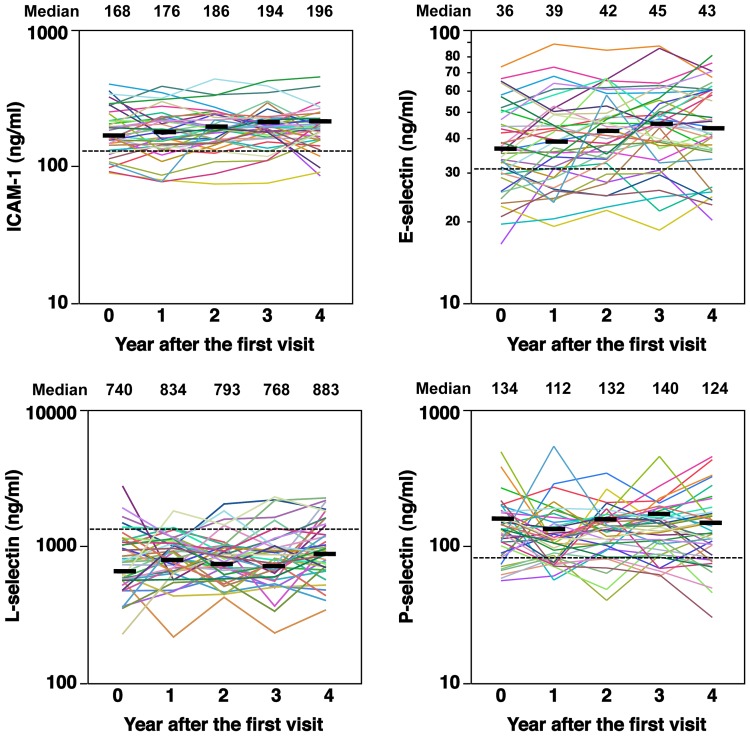
Longitudinal change of serum adhesion molecule levels in each patient during the four years of the study. The horizontal dotted line indicates the median value in healthy controls. The horizontal bar at each time point indicates the median value.

The yearly changes in serum adhesion molecule levels for each case are shown in Figure 2. The dotted horizontal lines indicate median values of healthy controls. Overall, the levels of each adhesion molecule in the serum showed considerable variations in each patient. However, the median values of ICAM-1, E-selectin, L-selectin, and P-selectin measured did not change significantly over time (Figure 2). Nonetheless, the median levels of ICAM-1, E-selectin, and L-selectin tended to slightly increase during the course. The variation in adhesion molecule levels over time was not significantly associated with the variation of the dose of steroid, MRSS, %VC, and HAQ-DI during the four years’ course of the study (data not shown).

### Association between Each Adhesion Molecule Level and Subsequent Severity of Clinical Features

We evaluated if baseline serum adhesion molecule levels are associated with baseline and subsequent clinical features of SSc by univariate analysis. Baseline serum ICAM-1 levels were significantly inversely associated with %VC values at baseline (r = -041, p<0.05) and subsequent every year until 4 years (Table 2). Additionally, serum ICAM-1 levels at the first year were significantly negatively associated with %VC values at the third and fourth year (Table 3). Similarly, ICAM-1 levels at the second year were significantly inversely associated with %VC at the second, third, and fourth year. ICAM-1 levels of the third year were significantly negatively correlated with %VC of the third and fourth year. Baseline serum P-selectin levels were significantly associated with HAQ-DI values at baseline (r = 0.51, p = 0.001) and the first, second, and fourth year (Table 2). However, serum P-selectin levels at the subsequent years did not significantly associate with HAQ-DI at the same time or subsequent years (Table 4). Otherwise, no significant associations between serum levels of adhesion molecules and subsequent clinical features were found. These data indicate that serum level of ICAM-1 is a useful biomarker for estimating the current and subsequent respiratory dysfunction. Additionally, baseline serum P-selectin level may reflect the current and subsequent physical disability.

**Table 2:** The associations between baseline adhesion molecule levels and subsequent clinical parameters in patients with SSc.

	Baseline	1 yearfollow-up	2 yearfollow-up	3 yearfollow-up	4 yearfollow-up
Log_10_ (ICAM-1 (ng/ml)) (baseline) vs. MRSS (baseline∼4 year)	r = -0.12	r = −0.02	r = −0.02	r = −0.15	r = −0.072
	p = 0.48	p = 0.90	p = 0.90	p = 0.37	p = 0.66
Log_10_ (ICAM−1 (ng/ml)) (baseline) vs. %VC (baseline∼4 year)	r = −0.41*	r = −0.40*	r = −0.41*	r = −0.57**	r = −0.59**
	p = 0.019	p = 0.033	p = 0.036	p = 0.0027	p = 0.0009
Log_10_ (ICAM−1 (ng/ml)) (baseline) vs. HAQ-DI (baseline∼4 year)	r = 0.065	r = 0.027	r = −0.14	r = 0.11	r = 0.060
	p = 0.69	p = 0.87	p = 0.39	p = 0.49	p = 0.72
Log_10_ (E-selectin (ng/ml)) (baseline) vs. MRSS (baseline∼4 year)	r = 0.16	r = 0.12	r = 0.012	r = 0.012	r = 0.16
	p = 0.33	p = 0.46	p = 0.94	p = 0.94	p = 0.33
Log_10_ (E-selectin (ng/ml)) (baseline) vs. MRSS (baseline∼4 year)	r = −0.30	r = −0.30	r = −0.29	r = −0.06	r = −0.30
	p = 0.13	p = 0.25	p = 0.14	p = 0.77	p = 0.13
Log_10_ (E-selectin (ng/ml)) (baseline) vs. HAQ-DI (baseline∼4 year)	r = 0.12	r = 0.056	r = 0.12	r = 0.19	r = 0.012
	p = 0.94	p = 0.73	p = 0.48	p = 0.25	p = 0.94
Log_10_ (L-selectin (ng/ml)) (baseline) vs. MRSS (baseline∼4 year)	r = −0.06	r = −0.10	r = −0.12	r = −0.08	r = −0.18
	p = 0.57	p = 0.37	p = 0.27	p = 0.51	p = 0.17
Log_10_ (L-selectin (ng/ml)) (baseline) vs. %VC (baseline∼4 year)	r = −0.051	r = 0.049	r = −0.052	r = −0.16	r = −0.26
	p = 0.68	p = 0.76	p = 0.71	p = 0.32	p = 0.13
Log_10_ (L-selectin (ng/ml)) (baseline) vs. HAQ-DI (baseline∼4 year)	r = −0.13	r = −0.062	r = −0.12	r = −0.058	r = −0.07
	p = 0.21	p = 0.57	p = 0.26	p = 0.63	p = 0.63
Log_10_ (P-selectin (ng/ml)) (baseline) vs. MRSS (baseline∼4 year)	r = 0.14	r = 0.30	r = 0.13	r = 0.079	r = 0.23
	p = 0.39	p = 0.060	p = 0.43	p = 0.63	p = 0.15
Log_10_ (P-selectin (ng/ml)) (baseline) vs. %VC (baseline∼4 year)	r = −0.16	r = −0.20	r = 0.022	r = −0.13	r = −0.077
	p = 0.37	p = 0.47	p = 0.91	p = 0.53	p = 0.70
Log_10_ (P-selectin (ng/ml)) (baseline) vs. HAQ-DI (baseline∼4 year)	r = 0.51**	r = 0.52**	r = 0.54**	r = 0.31	r = 0.36*
	p = 0.0010	p = 0.0006	p = 0.0004	p = 0.058	p = 0.026

*p<0.05, **p<0.01.

**Table 3:** The associations between ICAM−1 levels and subsequent %VC in patients with SSc.

	%VC (baseline)	%VC (1 yearfollow-up)	%VC (2 yearfollow-up)	%VC (3 yearfollow-up)	%VC (4 yearfollow-up)
Log_10_ (ICAM−1 (ng/ml)) (baseline)	r = −0.41*	r = −0.40*	r = −0.41*	r = −0.57**	r = −0.59**
	p = 0.019	p = 0.033	p = 0.036	p = 0.0027	p = 0.0009
Log_10_ (ICAM−1 (ng/ml))(1 year follow-up)		r = −035	r = −0.36	r = −0.56**	r = −0.46**
		p = 0.080	p = 0.079	p = 0.0042	p = 0.014
Log_10_ (ICAM−1 (ng/ml))(2 year follow-up)			r = −0.43*	r = −0.58**	r = −0.50**
			p = 0.028	p = 0.0022	p = 0.0074
Log_10_ (ICAM−1 (ng/ml))(3 year follow-up)				r = −0.55**	r = −0.39*
				p = 0.0048	p = 0.040
Log_10_ (ICAM−1 (ng/ml))(4 year follow-up)					r = −0.30
					p = 0.12

*p<0.05, **p<0.01.

**Table 4:** The associations between P-selectin levels and subsequent HAQ-DI in patients with SSc.

	HAQ-DI (baseline)	HAQ-DI (1 year follow-up)	HAQ-DI (2 yearfollow-up)	HAQ-DI (3 year follow-up)	HAQ-DI (4 year follow-up)
Log_10_ (P-selectin (ng/ml)) (baseline)	r = 0.51**	r = 0.52**	r = 0.54**	r = 0.31	r = 0.36*
	p = 0.0010	p = 0.0006	p = 0.0004	p = 0.058	p = 0.026
Log_10_ (P-selectin (ng/ml))(1 year follow-up)		r = −0.18	r = −0.064	r = 0.018	r = −0.12
		p = 0.29	p = 0.70	p = 0.91	p = 0.49
Log_10_ (P-selectin (ng/ml))(2 year follow-up)			r = 0.015	r = −0.074	r = −0.25
			p = 0.92	p = 0.66	p = 0.12
Log_10_ (P-selectin (ng/ml))(3 year follow-up)				r = 0.25	r = 0.017
				p = 0.12	p = 0.92
Log_10_ (P-selectin (ng/ml))(4 year follow-up)					r = 0.018
					p = 0.92

*p<0.05, **p<0.01.

### Association between the Level of Each Adhesion Molecule and the Severity of Clinical Features Analyzed by Multiple Regression Analysis

Next, we utilized multiple regression analysis to evaluate the ability of serum adhesion molecule levels to predict clinical or laboratory factors such as MRSS, %VC, and HAQ-DI of patients four years after the first visit. Selected variables were as follows: each adhesion molecule level, anti-topoisomerase I Ab, anticentromere Ab, MRSS, %VC, presence of ILD, HAQ-DI, ESR, corticosteroid treatment, and cyclophosphamide treatment at the first visit. We performed stepwise regression analysis that specified the α level for either adding or removing a regression as 0.15. As a result, the multiple regression equation predicting the %VC of 4 year follow-up = 230.2+0.62×%VC of baseline+−60.1×log_10_ (serum ICAM−1 levels (ng/ml)) of baseline (R^2^ = 0.73, root mean square error (RMSE) = 12.1, p<0.0001, Table 5). Using our equation, we found that the %VC value at the fourth year was significantly associated with the %VC of baseline (p = 0.0001) and was significantly inversely associated with the initial ICAM levels (p = 0.015). Multi-colineality was not detected between independent factors (variance inflation factor (VIF) = 1.20). The multiple regression equation predicting the HAQ-DI of 4 year follow-up = −2.75+2.22×log_10_ (serum P-selectin levels (ng/ml)) of baseline+−0.0060×%VC of baseline +0.29×HAQ-DI (R^2^ = 0.41, RMSE = 0.345, p = 0.001, Table 6). Using our equation, we found that the HAQ-DI value of 4-year follow-up was significantly associated with P-selectin levels of baseline (p = 0.028). The HAQ-DI value at the fourth year tended to be negatively associated with the %VC of baseline (p = 0.057) and tended to be positively associated with the initial HAQ-DI (p = 0.10). Multi-colineality did not exist among independent factors (VIF = 1.05−1.41). MRSS at the fourth year was not significantly associated with any adhesion molecule levels or clinical factors of baseline.

**Table 5:** Factors predicting %VC of 4 year follow-up determined by multiple regression analysis.

	Estimate	Standard error	P value
Intercept	230.2	83.4	0.012
%VC of baseline	0.62	0.13	0.0001
Log_10_ (serum ICAM-1 levels of baseline) ng/ml	−60.1	22.7	0.015

The multiple regression equations predicting %VC of 4 year follow-up are as follows; %VC of 4 year follow-up = 230.2+0.62×%VC of baseline+−60.1×log_10_ (serum ICAM-1 levels (ng/ml) of baseline). R^2^ (determination coefficient) = 0.73, root mean square error = 12.1, p<0.0001.

**Table 6:** Factors predicting HAQ-DI of 4 year follow-up determined by multiple regression analysis.

	Estimate	Standard error	P value
Intercept	−2.75	1.62	0.099
Log_10_ (serum P-selectin levels of baseline) ng/ml	2.22	0.96	0.028
%VC of baseline	−0.0060	0.0030	0.057
HAQ-DI of baseline	0.29	0.17	0.100

The multiple regression equations predicting HAQ-DI of 4 year follow-up are as follows; HAQ-DI of 4 year follow-up = −2.75+2.22×log_10_(serum P-selectin levels (ng/ml) of baseline)+−0.0060×%VC of baseline +0.29×HAQ-DI of baseline. R^2^ (determination coefficient) = 0.41, root mean square error = 0.345, p = 0.001.

### Association between Each Adhesion Molecule Level and Subsequent Change of Clinical Parameters

Finally, we evaluated if baseline serum adhesion molecule levels are associated with subsequent percent change of clinical parameters by univariate analysis. However, baseline serum adhesion molecule levels were not significantly associated with the percent change of MRSS, %VC, and HAQ-DI values every year until 4 years (Table S1). Additionally, the percent changes of clinical parameters including MRSS, %VC, and HAQ-DI during 4-years were not significantly associated with any baseline adhesion molecule levels by multiple regression analysis (data not shown).

## Discussion

In this study, serum levels of ICAM-1, E-selectin, and P-selectin were significantly elevated in early SSc patients with diffuse skin sclerosis and/or ILD. By contrast, serum L-selectin levels were significantly reduced in these SSc patients. In this multicentre, longitudinal, prospective study, a multiple regression equation was defined to predict symptoms four years after initial diagnosis using baseline serum levels of four adhesion molecules and multiple clinical or laboratory factors presenting at the time of the first visit. Our findings suggest that elevated serum ICAM-1 levels are useful to predict the subsequent respiratory dysfunction. Furthermore, serum P-selectin levels at baseline may reflect the subsequent physical disability.

Our findings indicate that serum ICAM-1 levels were inversely associated with the current and subsequent respiratory functions in patients with early SSc. Elevated serum levels of ICAM-1 measured in early SSc patients in our study are consistent with previous several reports investigated in SSc patients [20]-[22] or dcSSc patients [23]. In one of those studies, circulating ICAM-1 levels were especially elevated in patients with diffuse rapidly progressive disease or digital ulcers [20]. In one report, serum levels of ICAM-1, P-selectin, and to a lesser degree, E-selectin correlate well with their in situ expression and with clinical disease activity [21]. Another study demonstrated that ICAM-1 levels were significantly higher in dcSSc patients and were correlated with the presence of contracture of phalanges, pulmonary fibrosis, joint involvement, and increased erythrocyte sedimentation rate [22]. In this study, we investigated the association between ICAM-1 levels and clinical features focused on early SSc patients with diffuse skin sclerosis and/or ILD in larger and multicenter population. As a result, serum ICAM-1 levels were specifically inversely associated with respiratory dysfunction.

Among various adhesion molecules, ICAM-1 has been most thoroughly investigated in the pathogenesis of SSc [24]. ICAM-1 is induced through IL-1β, IFN-γ, and TNF-α and initiates the binding of leukocytes to endothelium. Several previous studies have shown that SSc fibroblasts exhibit increased surface ICAM-1 expression, suggesting an augmented potential for binding to T cells [25]. Another study demonstrated that ICAM-1 and vascular cell adhesion molecule (VCAM)-1 have important roles in the retention of myeloid cells in the skin of SSc patients [26], [27]. In tight-skin 1 mouse, a genetic model of skin fibrosis, it has been demonstrated that ICAM-1 expression contributes to the development of skin fibrosis, especially via ICAM-1 expressed on skin fibroblasts [28]. ICAM-1 deficiency ameliorates lung fibrosis induced by intratracheal bleomycin administration [29]. A recent study has shown that L-selectin and ICAM-1 regulate Th2 and Th17 cell accumulation in the skin and lungs, leading to the development of fibrosis in a bleomycin-induced fibrosis model [30]. These previous reports indicate that ICAM-1 is contributing to the development of inflammation and fibrosis in SSc via inducing the infiltration and activation of leukocytes. Furthermore, increased circulating ICAM-1 may be reflecting the vascular activation and inflammation in SSc. This may be the reason why serum ICAM-1 levels are highly associated with current and subsequent respiratory dysfunction.

Circulating ICAM-1 has been considered as the result of proteolytic cleavage of cell-bound ICAM-1 close to the cell membrane [31], [32]. ICAM-1 cleavage is regulated by tumor necrosis factor-α-converting enzyme and multiple kinases, including mitogen-activated protein kinase, S locus receptor kinase, and phosphoinositide 3-kinase pathways [33], [34]. There are some reports demonstrating the critical roles of these enzymes in SSc patients or animal model of SSc [35]-[38]. Soluble ICAM-1 is functionally active and retains the ability to inhibit leukocyte-endothelial cell interaction [39], [40]. On the other hand, soluble ICAM-1 has also been reported to promote angiogenesis [41] and induce the production of TNF-α, IFN-γ, IL-6, and macrophage inflammatory protein-2 [42], [43]. Thus soluble ICAM-1 may also have proinflammatory potential.

In the current study, serum P-selectin levels were found to be increased, consistent with previous reports [21], [44], [45]. However, another previous study showed normal levels of P-selectin in SSc [46]. Since our population was selected for early active SSc, serum P-selectin levels are likely elevated at least at early active stage. The baseline P-selectin levels were significantly associated with HAQ-DI at the fourth year as determined by multiple regression analysis. Recently, we reported that baseline serum CXCL8 (IL-8) levels were significantly associated with subsequent HAQ-DI in early SSc patients [47]. Therefore, we compared the utility between P-selectin and CXCL8 for predicting the subsequent HAQ-DI by multiple regression analysis in the current population. As a result, P-selectin was more useful serum indicator of subsequent HAQ-DI (data not shown). The roles of P-selectin in the pathogenesis of SSc remain unclear. In lung fibrosis mouse model induced by intratracheal bleomycin administration, P-selectin deficiency did not significantly affect the fibrosis of lungs [48]. On the other hand, another study showed that the loss of P-selectin augmented the fibrosis of both skin and lungs induced by intracutaneous bleomycin injection [30].

We detected that serum E-selectin levels are also significantly elevated in SSc patients in consistent with previous studies [46], [49]-[51]. Although serum E-selectin levels were significantly associated with the presence of pulmonary fibrosis in a previous study [50], we could not find any significant association with clinical features in our population selected as those with early SSc patients with diffuse skin sclerosis and/or ILD.

Previous findings regarding serum L-selectin levels were not consistent. Significantly reduced levels of serum L-selectin have been reported in patients with SSc [52]. In a recent report, serum L-selectin levels were reduced and were negatively associated with skin damage in patients with dcSSc [53]. By contrast, serum levels of L-selectin were significantly elevated in patients with SSc in another study [54]. Another group reported that the levels were not significantly different between SSc patients and normal controls [45]. However, our multicentre, larger studies indicate that serum L-selectin levels are decreased in early SSc patients with diffuse skin sclerosis and/or ILD. Serum L-selectin levels have been known to increase during acute inflammatory conditions as a result of shedding from activated leukocytes and/or leukocytes transmigrating endothelial cells. Although we can not explain why our patients with SSc showed reduced serum L-selectin levels, chronic inflammation such as chronic heart or renal diseases likely results in downregulation of leukocyte expression of cell-surface L-selectin and thus lower circulating L-selectin levels [55], [56].

Some limitations exist in this study. The population of longitudinal study is relatively small. Additionally, this is an observational study and, therefore, the treatment protocol is heterogeneous. Nonetheless, our data indicate that serum levels of ICAM-1 and P-selectin may be useful for predicting the subsequent severity of ILD and physical dysfunction, respectively. The predictive biomarkers are generally important if they predict the rates of change in the investigated outcomes rather than their absolute levels. However, the association between serum levels of adhesion molecules and the rates of change of investigated outcomes were not significant. Therefore, this study indicates that serum levels of ICAM-1 and P-selection are useful to predict the subsequent severity of respiratory dysfunction and physical disability, respectively, but not the subsequent rate of their change. Further longitudinal studies in a larger population will be needed to confirm the utility of these adhesion molecules as prognostic indicators in SSc patients.

## Supporting Information

Table S1 The associations between baseline adhesion molecule levels and subsequent percent change of clinical parameters in patients with SSc.
(DOCX)
