# Supplementary material for: Serum Adhesion Molecule Levels as Prognostic Markers in Patients with Early Systemic Sclerosis: A Multicentre, Prospective, Observational Study
Source: PLoS One. 2014 Feb 6;9(2):e88150. doi: 10.1371/journal.pone.0088150 (PMC3916412; doi:10.1371/journal.pone.0088150)
Supplement: Table S1 — The associations between baseline adhesion molecule levels and subsequent percent change of clinical parameters in patients with SSc. (DOCX) [file pone.0088150.s001.docx]

**Supporting information**

Table S1. The associations between baseline adhesion molecule levels and subsequent percent change of clinical parameters in patients with SSc

|  | Baseline~1 year | Baseline~2 year | Baseline~3 year | Baseline~4 year |
| --- | --- | --- | --- | --- |
| Log_10_ (ICAM-1 (ng/ml)) (baseline) vs. change (%/year) of MRSS (baseline~4 year) | r=0.081 | r=0.11 | r=-0.0021 | r=0.094 |
|  | p=0.61 | p=0.50 | p=0.99 | p=0.55 |
| Log_10_ (ICAM-1 (ng/ml)) (baseline) vs. change (%/year) of %VC (baseline~4 year) | r=-0.20 | r=-0.15 | r=-0.23 | r=-0.23 |
|  | p=0.44 | p=0.51 | p=0.29 | p=0.27 |
| Log_10_ (ICAM-1 (ng/ml)) (baseline) vs. change (%/year) of HAQ-DI (baseline~4 year) | r=-0.051 | r=-0.23 | r=0.046 | r=0.0050 |
|  | p=0.75 | p=0.16 | p=0.29 | p=0.81 |
| Log_10_ (E-selectin (ng/ml)) (baseline) vs. change (%/year) of MRSS (baseline~4 year) | r=0.099 | r=0.054 | r=-0.014 | r=0.079 |
|  | p=0.53 | p=0.75 | p=0.93 | p=0.61 |
| Log_10_ (E-selectin (ng/ml)) (baseline) vs. change (%/year) of %VC (baseline~4 year) | r=-0.27 | r=-0.20 | r=-0.014 | r=-0.37 |
|  | p=0.30 | p=0.37 | p=0.95 | p=0.054 |
| Log_10_ (E-selectin (ng/ml)) (baseline) vs. change (%/year) of HAQ-DI (baseline~4 year) | r=-0.076 | r=0.098 | r=0.13 | r=0.048 |
|  | p=0.63 | p=0.55 | p=0.41 | p=0.82 |
| Log_10_ (L-selectin (ng/ml)) (baseline) vs. change (%/year) of MRSS (baseline~4 year) | r=0.15 | r=0.017 | r=0.10 | r=0.078 |
|  | p=0.35 | p=0.92 | p=0.52 | p=0.62 |
| Log_10_ (L-selectin (ng/ml)) (baseline) vs. change (%/year) of %VC (baseline~4 year) | r=-0.16 | r=0.17 | r=0.15 | r=0.47 |
|  | p=0.55 | p=0.45 | p=0.51 | p=0.18 |
| Log_10_ (L-selectin (ng/ml)) (baseline) vs. change (%/year) of HAQ-DI (baseline~4 year) | r=0.17 | r=0.095 | r=0.19 | r=0.33 |
|  | p=0.27 | p=0.57 | p=0.23 | p=0.11 |
| Log_10_ (P-selectin (ng/ml)) (baseline) vs. change (%/year) of MRSS (baseline~4 year) | r=0.13 | r=0.021 | r=-0.12 | r=0.023 |
|  | p=0.43 | p=0.89 | p=0.47 | p=0.88 |
| Log_10_ (P-selectin (ng/ml)) (baseline) vs. change (%/year) of %VC (baseline~4 year) | r=-0.039 | r=-0.15 | r=-0.078 | r=-0.11 |
|  | p=0.89 | p=0.52 | p=0.72 | p=0.59 |
| Log_10_ (P-selectin (ng/ml)) (baseline) vs. change (%/year) of HAQ-DI (baseline~4 year) | r=-0.089 | r=0.14 | r=-0.16 | r=0.16 |
|  | p=0.57 | p=0.39 | p=0.31 | p=0.46 |
